# Targeting the stringent response alters gene expression and extracellular vesicle RNA in *Staphylococcus aureus*

**DOI:** 10.1128/spectrum.00359-26

**Published:** 2026-06-15

**Authors:** Sarah C. Mansour, Samuel J. T. Wardell, Arjun S. Baghela, Travis Blimkie, Erin Gill, Susana K. Straus, Reza Falsafi, Daniel Pletzer, Robert E. W. Hancock

**Affiliations:** 1 Centre for Microbial Diseases and Immunity Research, Department of Microbiology and Immunology, University of British Columbia 8166, Vancouver, Canada; 2 Department of Microbiology and Immunology, Faculty of Biomedical Sciences, University of Otago 2495, Dunedin, New Zealand; 3 Department of Chemistry, University of British Columbia 8166, Vancouver, British Columbia, Canada; Forschungszentrum Jülich GmbH, Jülich, Germany

**Keywords:** extracellular vesicles, antimicrobial peptides, attenuate virulence, antibiotic tolerance, DJK-5, MRSA

## Abstract

*Staphylococcus aureus* relies on the (p)ppGpp-mediated stringent response to adapt to nutrient limitation and other environmental stress, with important consequences for metabolism, antibiotic tolerance, and virulence. In this study, we identified that disruption of (p)ppGpp synthesis altered the small RNA (sRNA) cargo of extracellular vesicles (EVs) produced by *S. aureus*. We characterized EV-associated sRNAs from wild-type and stringent response mutant (*rsh*_syn_) strains, revealing the presence of SprX2 in mutant EVs. The *rsh*_syn_ mutant also showed modestly enhanced survival under high concentrations of vancomycin and oxacillin in late stationary phase. Transcriptomic analysis under mupirocin-induced amino acid limitation demonstrated broad ppGpp-dependent remodeling of gene expression, including changes in amino acid biosynthesis, translation-associated functions, adhesion factors, and virulence-associated genes. Treatment with the synthetic cationic peptide DJK-5 reduced intracellular ppGpp levels, suppressed the expression of genes involved in adhesion (*fnbA*, *fnbB*, *clfB*, *emp*), cytolytic toxins (*psmβ1*, *psmβ2, hla*, *lukED*), and exoenzymes (*lip*, *geh*, *sspA*, *aur*, *sspB*), and reduced methicillin-resistant *S. aureus* (MRSA)-mediated toxicity toward eukaryotic cells. These findings suggest that targeting the stringent response impairs both intracellular regulatory networks and EV-mediated signaling, offering a promising approach to attenuate *S. aureus* virulence and enhance efficacy against resistant infections.

**IMPORTANCE:**

The stringent response helps *Staphylococcus aureus* survive nutrient stress, adapt its metabolism, and regulate virulence. This study shows that disrupting this pathway not only alters intracellular gene expression but is also associated with changes in the small RNA (sRNA) cargo of extracellular vesicles. In addition, the synthetic peptide DJK-5 weakens the pathogen’s ability to regulate biofilm formation, its virulence, and reduces its communication through small RNA molecules, which are critical for stress adaptation and infection. These findings introduce an approach to weakening bacterial defenses and suggest that interfering with stress responses could enhance the effectiveness of existing antibiotics against antibiotic-resistant bacteria, offering a promising strategy for combating bacterial infections and reducing antibiotic resistance.

## INTRODUCTION

*Staphylococcus aureus* is a human pathogen that causes a wide spectrum of diseases, ranging from superficial skin infections (e.g., boils, abscesses, and impetigo) to more life-threatening conditions, such as sepsis, pneumonia, osteomyelitis, toxic shock syndrome, and endocarditis (1). In addition, *S. aureus* has become resistant to major antibiotics, giving rise to methicillin-resistant *S. aureus* (MRSA) (2), as well as vancomycin-intermediate and vancomycin-resistant *S. aureus* (3, 4). Over the past few decades, *S. aureus* infections have emerged in the community, causing invasive infections in otherwise healthy individuals (5, 6). The pathogenicity of community-associated MRSA (CA-MRSA) has been attributed to its heightened expression of virulence factors, rapid growth rate, and acquisition of mobile genetic elements that increase its ability to persist during infection (7). The increased transmissibility of *S. aureus,* coupled with its ability to develop resistance to many antibiotics, makes it a serious public health threat that requires greater attention (8–10).

Despite the increase in antibiotic resistance, few new antibiotic classes have been developed in recent decades (11). For this reason, there has been an increasing interest in exploiting natural products as novel therapeutic agents. Antimicrobial peptides (AMPs), for example, are found in virtually all organisms as a first line of defense against invading microbes (i.e., bacteria, viruses, fungi, and protozoa) (12). While their direct antimicrobial activity is often reduced under physiological conditions (13, 14), AMPs still provide protection by promoting the recruitment of immune cells (15) and modulating inflammatory responses (16). In addition, certain AMPs and synthetic derivatives, known as anti-biofilm peptides, can inhibit and eradicate surface-associated biofilm communities (17–20). Among these, we previously demonstrated that the synthetic peptide DJK-5 impaired *S. aureus* biofilm formation, suppressed *S. aureus* cutaneous abscess formation, and synergized with traditional antibiotics (20, 21), including last-line antibiotics colistin and daptomycin (22). The precise mechanism of action of DJK-5 is yet to be determined. However, some of the activity of DJK-5 is thought to occur through membrane perturbation and peptide interference with the stringent stress response (SSR) (20, 23), although these are unlikely to be the only mechanisms of action.

The SSR is one of the most important adaptation mechanisms used by bacteria to survive in various environmental stress conditions (24–26). Environmental stress, such as amino acid deprivation, heat shock, and antimicrobial challenges, leads to the synthesis of guanosine tetraphosphate (ppGpp) and guanosine pentaphosphate (pppGpp), collectively known as (p)ppGpp (24). The accumulation of (p)ppGpp triggers major cellular reprogramming in bacteria, leading to the repression of energy-intensive processes, such as translation, lipid and cell wall biosynthesis, and transcription, while simultaneously activating various survival processes (25, 27–29). In many pathogens, including *S. aureus*, (p)ppGpp is required for biofilm initiation and maintenance, and bacterial mutants defective in the stringent response exhibit reduced or altered biofilm composition (18, 30, 31). In addition to impaired biofilm formation, SSR mutants often exhibit attenuated virulence in live infection models (20, 23, 32–34). We have previously demonstrated the importance of a fully functional stringent response in *S. aureus* (20) and *Pseudomonas aeruginosa* (23) cutaneous abscess formation.

In *S. aureus*, under amino acid starvation, (p)ppGpp production is mediated by the bi-functional synthase/hydrolase enzyme RSH (a RelA/SpoT homolog) (24, 27). The RSH enzyme possesses both synthase and hydrolase domains, as well as a C-terminal sensory domain that responds to environmental stimuli, such as the accumulation of uncharged transfer RNA (tRNA) (26). These uncharged tRNAs activate the *rsh* synthetase domain at the ribosome, leading to rapid production of (p)ppGpp. A well-characterized *rsh* synthase-domain mutant, previously described by Geiger et al. (27), carries a three-amino acid deletion that abolishes (p)ppGpp synthesis during amino acid starvation or mupirocin treatment. This mutant has been instrumental in defining the role of Rsh in *S. aureus* amino acid biosynthesis, metabolism, virulence, and stress adaptation.

*S. aureus* possesses two other putative ppGpp synthases, RelP and RelQ, which are active under cell wall stress conditions, including exposure to β-lactams (35). Studies by Corrigan et al*.* (36) showed that (p)ppGpp binds with high affinity to various cellular GTPases involved in ribosome assembly, directly impairing protein synthesis. In addition, ppGpp can induce numerous regulatory changes by indirectly modulating the repressor function of CodY (29, 37). CodY is a transcriptional regulator that targets more than 200 genes involved in metabolism, virulence, and environmental adaptation (29). CodY directly interacts with GTP and branched-chain amino acids (BCAAs), detecting changes in nutrient availability (37). For example, under normal nutrient conditions, GTP and BCAAs associate with CodY, increasing its affinity to various DNA target sites and, in turn, repressing transcription (37). Under stringent stress conditions, however, GTP pools are depleted to synthesize ppGpp, leading to derepression of CodY-targeted genes and increased bacterial virulence (29, 38).

While numerous studies have investigated the role of the SSR in *S. aureus* virulence, persistence, and physiology (39–41), as well as its impact on log-phase mRNA turnover through the SOS response (26, 42, 43), the roles of small RNAs (sRNAs) in activation and signaling remain underexplored. Small RNAs have been implicated in regulating gene expression under various stress conditions, including oxidative stress, hyperosmotic stress, pH fluctuations, and cold shock, as well as in controlling virulence (44). Several sRNAs are regulated by CodY during both the adaptive and stringent responses (37, 44–46). As such, they likely play an important role in the SSR and may even facilitate cell-to-cell communication under stress conditions. Notably, sRNAs have been implicated in cell-to-cell signaling through bacterial extracellular vesicles (EVs) (47, 48), suggesting a potential mechanism for coordinating stress responses across bacterial populations.

The exact consequences of pharmacologically impeding the SSR and its effect on extracellular bacterial communication remain poorly understood. In this study, we first identified changes in the EV payload of an SSR mutant during amino acid deprivation. We then examined the effect of DJK-5 on *S. aureus* targets regulated by the stringent response. DJK-5 altered intracellular ppGpp levels and bound to and co-precipitated with ppGpp. In addition, during starvation conditions, DJK-5 interfered with the induction of various hallmark stringent response genes, consistent with an observed peptide-induced protection of red blood cells and epithelial cells against MRSA. These results suggest that impairing the stringent response affects bacterial communication and virulence-associated processes.

## MATERIALS AND METHODS

### Bacterial strains

We used the *S. aureus* wild-type HG001 and the *rsh* synthase-domain mutant (HG001-86) (27) and methicillin-resistant *S. aureus* USA300 LAC (49) in this study.

### RNA isolation and RNA-Seq from *S. aureus* WT and RSH-synthase mutant

Cultures of HG001 *S. aureus* and its stringent response mutant *rsh*_syn_ were grown in 50 mL of tryptic soy broth (TSB). When cultures reached an OD_600_ of 0.5, they were split into two 25 mL cultures; one flask received 0.5 μg/mL mupirocin (Sigma-Aldrich) and the other was left untreated. After 30 min of treatment, 1.5 mL of bacterial culture was stabilized in 3 mL of RNAprotect Bacteria Reagent (Qiagen). After 5 min of incubation, the RNAprotect was removed following centrifugation, and the bacterial pellet was either stored at −80°C or processed directly for RNA isolation. Bacterial pellets were resuspended in RLT supplemented with β-mercaptoethanol and placed into Lysing Matrix B (MP Biomedicals) tubes. Cell lysis was carried out by homogenizing the tubes using the Bead Ruptor24 (OMNI International) for 1 min at 6.45 m/s. Subsequently, lysates were placed on ice for 5 min and then centrifuged at 13,000 × *g* for 15 min at 4°C to remove cell debris. Supernatants were added to ice-cold 100% ethanol and then added to RNAeasy mini columns. RNA cleanup was carried out using the Qiagen RNAeasy Mini Kit (Qiagen) according to the manufacturer’s protocol. RNA was eluted in nuclease-free water, and RNase inhibitor was added before samples were treated with DNAse to eliminate genomic DNA residues.

Before sequencing, 16S and 23S rRNA were removed from RNA samples using the Ribo-Zero rRNA removal kit (Illumina). The remaining mRNA was recovered by ethanol precipitation before being converted to a library of cDNA fragments by second-strand synthesis and enzymatic fragmentation. Subsequently, adapters were ligated onto library fragments to enable indexing and sequencing using the next-generation Illumina HiSeq 2500 sequencing platform at the UBC Sequencing and Bioinformatics Consortium. Sequence quality was assessed using FastQC v0.11.5 and MultiQC v0.8.dev0. Sequence reads generated from RNA-Seq were mapped and aligned to the annotated reference genome (NCTC8325, assembly ASM1342v1) using Bowtie2 v2.4.1 (50). The resulting BAM files were then converted to SAM files using SAMtools v1.15 (51), and read count tables were generated with HTSeq-count (52). Differential expression analysis between treatment conditions and genotypes was performed using DESeq2 v1.40.2 (53). RNA-Seq was performed on three independent biological replicates and using a fold-change cut-off of ≥1.5 with a FDR-adjusted *P* value cut-off of ≤0.05. Pathway enrichment analysis was carried out using signature over-representation analysis (54), an R package that identifies the most relevant pathways enriched in a data set based on genes or gene pairs specific to a single pathway. The KEGG repository (55) was used for pathway and gene annotations.

### Extracellular vesicle isolation and sRNA sequencing

Cultures of HG001 *S. aureus* wild type and its stringent response mutant *rsh_syn_* (*n* = 3 for each) were grown in 100 mL TSB until late stationary phase to ensure amino acid starvation (24 h). Small RNA was isolated from crude EV preparations (48). Briefly, cultures were centrifuged to remove whole bacterial cells at 3,000 × *g* for 40 min at 4°C. The resulting supernatant was filtered using consecutive 0.4 and 0.2 μm filters. The filtrate was concentrated using a 10 kDa filter (Amicon Ultra-15 centrifugal filter unit), centrifuged 6,000 × *g* for 30 min each loading. Concentrated retained supernatant (typically 200–300 μL) was treated with DNase (Qiagen) and RNase A (Qiagen) to remove nucleic acids not protected from vesicles. The supernatant was also plated onto TSB agar to ensure no viable bacterial cells passed through the extraction. RNA was extracted from samples using the Qiagen miRNeasy kit following the manufacturer’s instructions, which included another on-column DNAse treatment. Sequencing of RNA was carried out using NextFlex small RNA-seq lib prep kit 1 × 100 bp SR on an Illumina NextSeq 2000 at the Otago Genomics Facility (University of Otago, New Zealand).

Sequencing quality was assessed using FastQC v0.11.5 and MultiQC v1.13.dev0. Sequencing adapters (3′) and four arbitrary bases added 3′ and 5′ by the small RNA-seq library prep kit were trimmed using cutadapt 4.1 (56). Trimmed reads were mapped to *S. aureus* HG001 (GCF_001900185.1) using bowtie2 v2.4.5 (50) with the “--local” flag enabled to assess bacterial origin and transcript composition. For descriptive annotation of EV-associated sRNAs, trimmed reads were queried against a curated BLAST database derived from the *Staphylococcal* regulatory RNAs database (57), which included 2,776 predicted and verified sRNA sequences in five *S*. *aureus* reference strains (JKD6008, N315, NCTC8325, Newman, and USA300). Each sample was also converted from fastq to fasta format and blasted against these sRNA sequences using blastn v2.6.0+ using an e-value cut-off of 10^−6^. Counts for each unique sRNA were determined for wild-type HG001 and the stringent response mutant *rsh_syn_*, and sRNA with fewer than three unique reads were excluded from the analysis. Because the database contains homologous sRNAs from multiple reference strains, hits were retained as candidate annotations and summarized descriptively rather than treated as formal differential-expression measurements. Sequencing yielded, on average, 3.65 million reads per sample.

### Antibiotic tolerance to vancomycin and oxacillin

Both the wild type and mutant were grown (37°C, 250 rpm) to late stationary phase in TSB (20 h) and subsequently treated with vancomycin (250 μg/mL, 100× minimal inhibitory concentration [MIC]), oxacillin (25 μg/mL, 100× MIC), DJK-5 (32 μg/mL), or a combination. Bacterial survivors (colony-forming units, CFU/mL) were determined after another 24 h via serial dilution plating. The predicted additive effect (PAE) between DJK-5 and the conventional antibiotics was determined by the sum of CFU log reduction obtained for each single treatment, and a Mann-Whitney test was performed to compare PAE to the CFU counts obtained by the drug combination; synergy: *P* ≤ 0.05 (58).

### Peptide synthesis

DJK-5 (VQWRAIRVRVIR-NH_2_; all D amino acids) was synthesized by CPC Scientific using solid-phase 9-fluorenylmethoxy carbonyl (Fmoc) chemistry and purified to >95% purity using reverse-phase high-performance liquid chromatography.

### 31-Phosphorus NMR

To examine the ppGpp levels in *S. aureus* after being subjected to DJK-5, *S. aureus* HG001 was grown in 200 mL of TSB broth to late stationary phase (ppGpp-inducing conditions). Subsequently, 50 mL of bacterial suspension was removed and placed into a separate 250 mL Erlenmeyer flask and left untreated or treated with 64 or 96 μg/mL of DJK-5 for 60 min. The cells from each sample were then centrifuged for 20 min, 2,000 × *g*. Each sample was resuspended in 400 μL deionized H_2_O and then added to 500 μL of 13 M formic acid and 100 μL dH_2_O. The samples were subjected to three rounds of freezing and thawing using liquid nitrogen. The thawed samples were centrifuged at 13,000 × *g* at 4°C, and 500 μL of supernatant (containing extracted nucleotides) was used for ^31^phosphorus nucleic magnetic resonance (^31^P-NMR) spectroscopy analysis. Spectra were acquired as previously described (18). Trimethyl phosphate was used as an internal control to quantify ppGpp signals. Experiments were repeated three times on different days using one sample/treatment group.

### RNA isolation and quantitative real-time PCR of mupirocin- and DJK-5-treated cultures

Cultures of HG001 *S. aureus* were grown in 100 mL of TSB, and at an OD_600_ of 0.5, they were split into four 20 mL cultures: two flasks received 0.5 μg/mL mupirocin (Sigma-Aldrich), and the other two were left untreated. After 30 min of incubation, 32 μg/mL of DJK-5 was added to one mupirocin-treated and one mupirocin-untreated flask, and the flasks were incubated for an additional 60 min. After this time, 1.5 mL of bacterial culture from each of the four flasks was stabilized in 3 mL of RNAprotect Bacteria Reagent (Qiagen), and RNA isolation was performed as described above.

Using 5 ng/sample of RNA, cDNA synthesis and PCR amplification were carried out using the qScript One-Step SYBR Green qRT-PCR Kit (QuantaBio) in combination with a Roche LightCycler 96 instrument, according to the manufacturer’s protocol. The sequences for the specific primers used are found in Table S1. Experiments were conducted in triplicate, and quantification of mRNA transcripts was performed using linear regression via the *pcr* package in R as previously described (59) using *pyk* as the housekeeping gene. The *P* values represent the statistical significance of the differences in Δ*Ct* values between the control and treatment groups.

### LDH release from human bronchial epithelial cells

The human bronchial epithelial cell (HBEC) line 16HBE14o- was maintained in Gibco Minimum Essential Media (Thermo Fisher Scientific) supplemented with 10% fetal bovine serum (Thermo Fisher Scientific), 2 mM L-glutamine (Thermo Fisher Scientific), and 100 U/mL penicillin/streptomycin (Thermo Fisher Scientific) at 37°C in 5% CO_2_. MRSA USA300 was grown with 0.5, 1, 2, or 4 μg/mL DJK-5 overnight in TSB broth. Bacterial supernatants were collected by centrifuging overnight samples at 13,000 × *g* for 5 min and subsequently filtering through a 0.2 μm filter (Thermo Fisher Scientific) to remove bacteria. Bacterial supernatants (75 μL) were used to treat confluent monolayers of HBE cells (8 × 10^4^ cells/well) for 1 h at 37°C in 5% CO_2_. HBE cells treated with 2% (vol/vol) Triton X-100 served as a positive control, and TSB-treated HBE cells served as a negative control. After 1 h, supernatants were collected to quantify LDH release using the cytotoxicity detection kit (Roche) according to the manufacturer’s instructions. All experiments were repeated three times.

### Red blood cell hemolysis

Venous blood was collected from healthy volunteers (following University of British Columbia ethics guidelines and certification) and stored in sodium heparin blood collection tubes (BD Vacutainer, VWR). RBCs were isolated from whole blood by centrifugation at 500 × *g* for 10 min, washed twice with 1× PBS and resuspended in 1 mL of Alsever’s solution (Sigma-Aldrich). USA300 was grown overnight with 0.5, 1, 2, or 4 μg/mL DJK-5 in TSB broth. Bacterial supernatants were collected by centrifuging overnight samples at 13,000 × *g* for 5 min. Bacterial supernatants (50 μL) were incubated with 1% RBC for 1 h at 37°C to make a final concentration of 200 μL. RBCs treated with 2% (vol/vol) Triton X-100 served as a positive control, and RBCs treated with TSB served as a negative control. After 1 h, RBCs were centrifuged at 500 × *g* for 10 min, and the hemoglobin content in the supernatants was measured using a microplate reader at 450 nm (reference 630 nm). Percentage hemolysis was calculated using the following formula: (ΔOD_sample_ – ΔOD_negative control_)/(ΔOD_positive control_ – ΔOD_negative control_) × 100. All experiments were repeated three times, each time with a different blood donor.

### Statistical analysis

Statistical evaluations were performed using GraphPad Prism 7.0 (GraphPad Software, La Jolla, CA, United States). All *P* values were calculated using one-way ANOVA, Kruskal-Wallis multiple-comparison test, followed by a Dunnett’s post hoc test. Data were considered significant when *P* values were below 0.01, as indicated.

## RESULTS AND DISCUSSION

**Fig 1:**
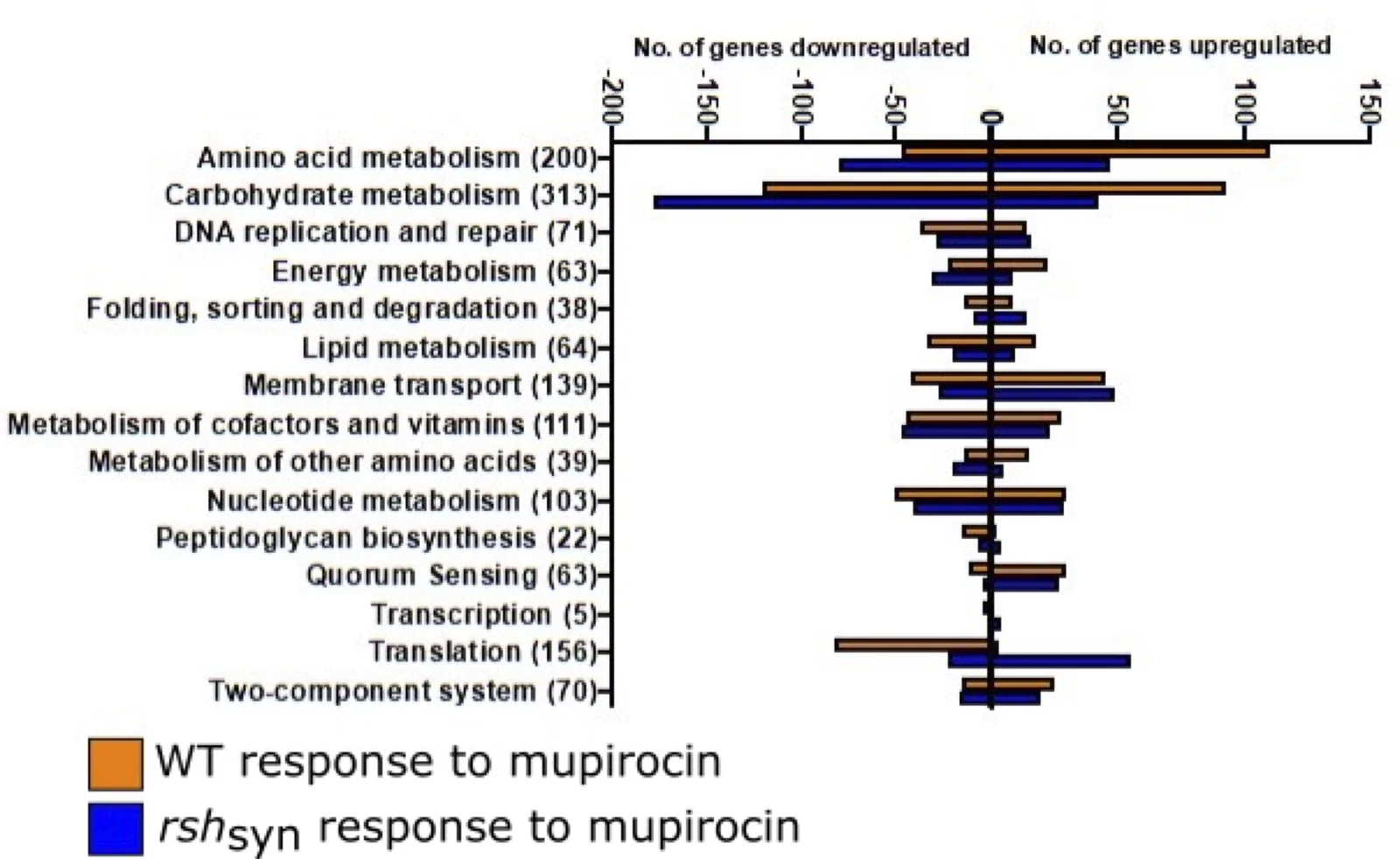
Induction of stringent stress response causes widespread transcriptional changes in both wild-type and stringent response lacking mutant. Differentially expressed genes from WT mupirocin vs WT (orange) and *rsh*_syn_ mupirocin vs *rsh*_syn_ (blue) comparisons were grouped into KEGG pathways. The number of significantly upregulated and downregulated genes in each respective pathway is displayed via the bars, and the total number of genes in each pathway is listed beside the pathway name in brackets.

The stringent response is a conserved mechanism of bacterial adaptation and tolerance, enabling bacteria to colonize, infect, and persist under adverse conditions (24, 26). It is also important for stress signaling in response to nutrient deprivation and for controlling cellular pathways, including transcription and pathogenesis (60). To provide transcriptional context for the ppGpp-dependent phenotypes examined below, we first profiled the responses of WT and *rsh*_syn_ (synthase mutant) cells to mupirocin-induced stringent stress. RNA-seq analysis showed that mupirocin triggered broad transcriptional remodeling in both strains, affecting 1,722 and 1,519 genes, respectively. In WT, the response was characterized by induction of amino acid biosynthetic/metabolic pathways and repression of translation-associated functions, whereas the response in the *rsh*_syn_ mutant was altered, consistent with a ppGpp-dependent stringent-response program (26, 27, 39, 42, 43) (Fig. 1). Full gene lists and enrichment analyses are provided in Text S1 and Tables S2 to S4.

### Amino acid limitation drove changes in sRNA payloads of EVs in the SSR mutant

Recent studies suggest that RNA within *S. aureus* EVs plays a crucial role in facilitating bacterial adaptation to stress (47, 48). Guided by transcriptional evidence for ppGpp-dependent remodeling of amino acid metabolism and related regulatory pathways (Text S1), we investigated the sRNA content in EVs from wild-type and stringent response mutant strains grown to stationary phase. This investigation was further motivated by evidence that EVs can carry and deliver tRNA fragments to eukaryotic host cells and our observation that nearly all tRNA-encoding genes were downregulated in the stringent response mutant (Text S1 and Tables S2 and S4). Based on these findings, we hypothesized that the SSR may influence the packaging of tRNA fragments and sRNA into EVs, thereby modulating bacterial adaptation to nutrient stress and potentially facilitating cell-to-cell communication or host interactions under adverse conditions.

Intriguingly, we found substantial differences in the diversity and abundance of bacterial sRNA species between WT bacteria and the SSR mutant (Table S5). To further determine the diversity and abundance of sRNA within each sample, unique reads that passed QC were searched against a database (57) containing 2,776 sRNA sequences from five different *S. aureus* species, including experimentally validated and computationally predicted sequences. In total, 67 predicted sRNA species were detected in the stringent response *rsh_syn_* mutant EVs, and 30 in the wild type (Table S5). Unsurprisingly, most unique matches were found in *in silico-*predicted unstudied sRNAs. However, seven sRNAs detected only in the wild type and 12 detected only in the mutant corresponded to previously described sRNA species (Table 1). Interestingly, one sRNA species detected only in the mutant was SprX2, a member of the small pathogenicity island RNA X (SprX) family. SprX-family sRNAs have been linked to glycopeptide susceptibility through repression of SpoVG (61), and SprX-family members have also been implicated in biofilm formation and autolysin regulation (62).

**TABLE 1:** Previously described sRNAs detected in late-stationary-phase EVs exclusively in either the wild-type or the *rsh_syn_* mutant

Name	RNA class/description	Putative function or regulatory role
Detected only in wild type		
*rsaOL*	Trans-encoded sRNA	Putative post-transcriptional regulation; specific targets unknown
*teg20*	Glucosamine-6-phosphate associated ribozyme	Glucosamine-6-phosphate sensing
*teg76*	Purine riboswitch	Purine-responsive cis-regulation
*sau59*	*Staphylococcus* sRNA sau-59	Possible condition-specific adaptation
*sprG4*	Type I toxin RNA/toxin-antitoxin component	Predicted toxin molecule
*sRNA263*	Ribosomal protein L20 leader	Ribosome biogenesis
*sRNA153*	*Staphylococcus* sRNA 35	Possible stress or host-adaptation role
Detected only in stringent response mutant *rsh_syn_*		
*sprX2*	SprX family trans-encoded sRNA	Post-transcriptional regulation linked to virulence
*teg1*	SAM riboswitch (S box leader)	Sulfur metabolism; SAM biosynthesis
*teg25*	SAM riboswitch (S box leader)	Sulfur metabolism; SAM biosynthesis
*teg51*	Purine riboswitch	Purine-responsive cis-regulation
*teg69*	T-box leader	Amino acid metabolism
*teg70*	T-box leader	Amino acid metabolism
*teg75*	SAM riboswitch (S box leader)	Sulfur metabolism and methylation
*tsr25*	*S. aureus* tsr25 small RNA	Possible serum/stress adaptation
*tsr33*	3′ UTR-associated small RNA	Possible 3′ UTR-mediated post-transcriptional regulation/function unknown
*sRNA264*	Lysine riboswitch	Lysine biosynthesis
*sprA1*	Type I toxin RNA (SprA1); encodes the toxic peptide PepA1	Toxin-antitoxin module/membrane-damaging toxin
*sprF1*	Type I RNA antitoxin (SprF1) of the SprG1/SprF1 module	Toxin neutralization/post-transcriptional toxin repression

**Fig 2:**
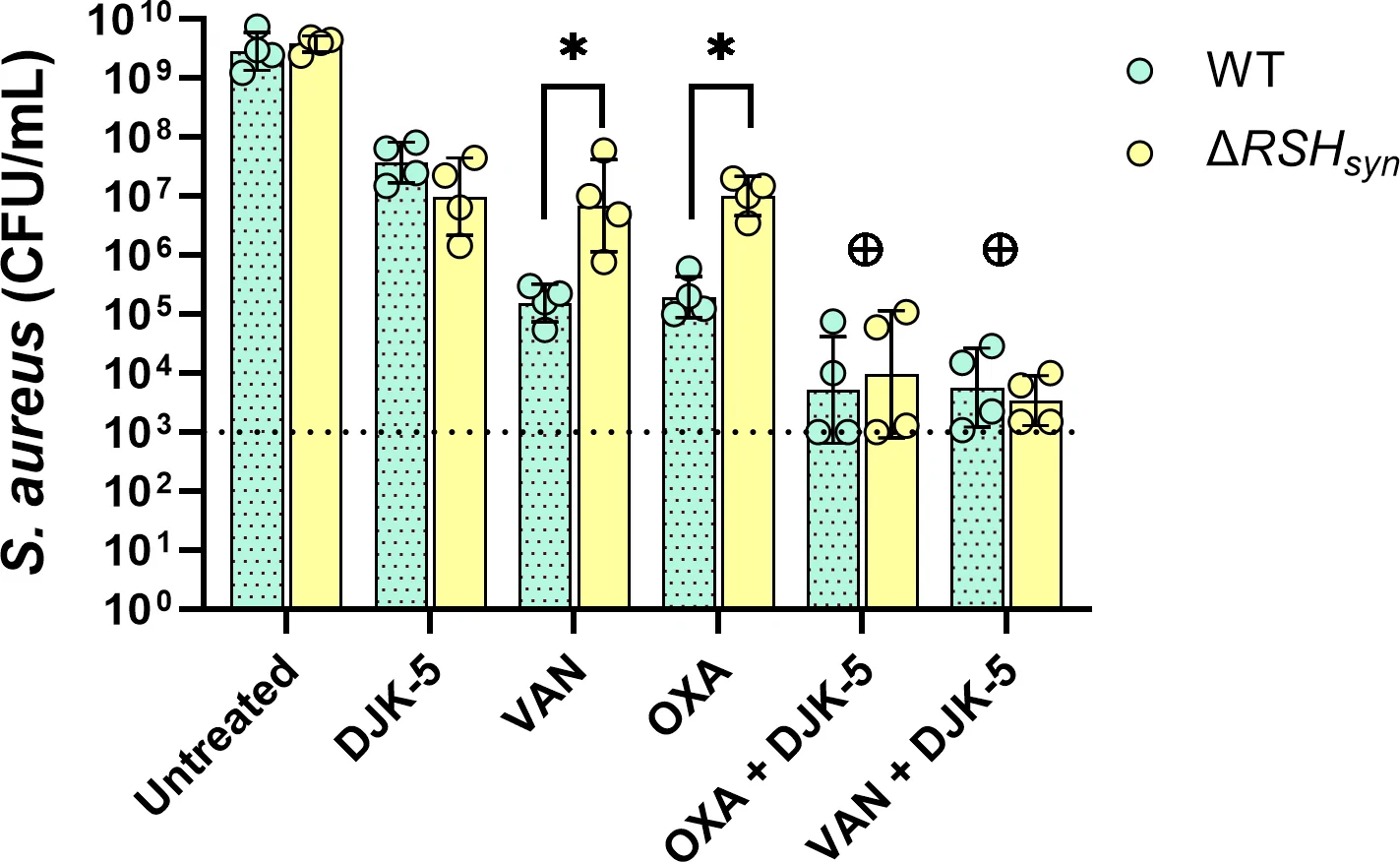
*S. aureus* HG001 wild-type and stringent response Δ*rsh_syn_* treatment. Both the wild type and mutant were grown to late stationary phase in TSB (20 h) and subsequently treated with vancomycin (250 μg/mL), oxacillin (25 μg/mL), DJK-5 (32 μg/mL), or a combination. Bacterial survivors (CFU/mL) were determined after an additional 24 h. *, *P* value < 0.05; two-way ANOVA, *n* = 4. Sum of CFU log reduction obtained for every single treatment, and a Mann-Whitney test was performed to compare PAE of the individual treatments to the CFU counts obtained by the drug combination ⊕, *P* ≤ 0.05 is synergistic.

We further examined the *rsh_syn_* mutant and WT in stationary phase. We found a modest increase in survival of the *rsh_syn_* mutant under high doses (100× MIC) of vancomycin and oxacillin compared to wild type in late stationary phase (Fig. 2). The *rsh_syn_* mutant showed enhanced survival to vancomycin and oxacillin of 46-fold (7 × 10^6^ vs 1.5 × 10^5^ CFU/mL) and 40-fold (1 × 10^7^ vs 2.5 × 10^5^ CFU/mL), respectively, compared to wild type. This contrasts with the current literature, which indicates that a functional stringent response machinery increases tolerance to some antibiotics (63 64 ). However, it should be stressed that no change was observed here in MIC between the WT and *rsh_syn_* mutant. Interestingly, this difference was overcome with the addition of the D-enantiomeric peptide DJK-5, which significantly synergized with vancomycin and oxacillin and allowed for comparable killing between wild-type and *rsh_syn_* mutant (Fig. 2).

Among the EV-associated sRNAs detected only in the *rsh_syn_* mutant, SprX2 was selected for closer discussion because it has been linked to regulators of cell aggregation and biofilm formation (62). SprX-family sRNAs repress SpoVG accumulation (61), and SpoVG, in turn, represses the regulation of *sasC* and extracellular DNA release, thereby modulating cell aggregation and biofilm-associated phenotypes. In our mupirocin-induced stringent stress transcriptome dataset, *sasC* was upregulated in the *rsh_syn_* mutant (2.2-fold; Tables S3 and S4), whereas *spoVG* was more highly expressed in the WT (2.1-fold; Tables S2 and S4), which is consistent with the possibility that SprX2 may contribute to the altered SpoVG-SasC regulatory axis in the mutant background (65, 66). However, because we did not test SprX2 genetically in the *rsh_syn_* background, this interpretation remains correlative. We therefore present the SprX2–SpoVG–SasC relationship as a working hypothesis rather than a validated mechanism (Fig. S1). Other altered EV-associated sRNAs may also contribute to the observed phenotype.

One other possible contributing factor might be changes in sRNA-mediated regulation of virulence genes. SprX-family sRNAs have been implicated in the regulation of virulence-associated genes in *S. aureus*. In particular, SprX1 has been shown to promote the expression of the cell wall-associated clumping factor B (*clfB*) and delta hemolysin (*hld*), while reducing the expression of antigen A (*isaA*) in the Newman background (67). Interestingly, we observed a similar pattern in our dataset: in the wild type treated with mupirocin to trigger the stringent response, *clfB* (1.8-fold) and *hld* (2.2-fold) were upregulated, whereas *isaA* was downregulated (−1.6-fold). This expression pattern was absent in the *rsh_syn_* mutant (Tables S2 to S4), suggesting that the SSR may be associated with SprX-mediated regulation of virulence genes.

EVs are increasingly recognized as mediators of intercellular communication and can influence neighboring bacteria, even across different species (68, 69). In this context, our findings suggest that disruption of the SSR, in addition to causing widespread intracellular transcriptional changes, may also alter the composition of EVs and their signaling capacity. However, we acknowledge that these associations remain correlative, and future targeted deletions and expression analyses will be required to test their mechanistic contribution to stringent-response-linked phenotypes.

### Stringent regulation of genes involved in colonization and virulence

Stress signals can trigger biofilm production in bacteria (70). Here, we further examined individual genes known to be involved in bacterial adhesion and biofilm formation (71) (Table 2). Attachment of *S. aureus* to surfaces is mediated in part by surface molecules known as MSCRAMMS (microbial surface component recognizing adhesive matrix molecules), which bind to human matrix molecules present in tissues and/or readily coat indwelling devices upon implantation. Amino acid deprivation upregulated the expression of numerous MSCRAMMS, including fibronectin binding protein genes *fnbA*, *fnbB*, *clfB,* and *emp* by 4.3-, 1.5-, 1.8-, and 1.9-fold, respectively (Table 2; Tables S2 and S4). Intriguingly, fibronectin-binding proteins A and B remained significantly upregulated in the *rsh_syn_* stringent response mutant.

**TABLE 2:** Adhesion and virulence genes dysregulated in the WT and *rsh*_syn_ mutant under stringent (mupirocin-treatment) conditions^*a*^

Gene	Loci	Name	Fold change mupirocin-treated WT	Fold change mupirocin-treated *rsh*_syn_
*fnbA*	SAOUHSC_02803	Fibronectin binding protein A	4.3	2.2
*fnbB*	SAOUHSC_02802	Fibronectin binding protein B	1.5	2.6
*clfB*	SAOUHSC_02963	Clumping factor B	1.8	1.0
*emp*	SAOUHSC_00816	Extracellular matrix protein	1.9	1.0
*lukD*	SAOUHSC_01954	Leukotoxin D	2.5	4.0
*lukE*	SAOUHSC_01955	Leukotoxin E	2.6	2.6
*hla*	SAOUHSC_01121	Hemolysin/alpha toxin	2.1	3.6
*lip*	SAOUHSC_03006	Lipase 1	5.5	1.2
*geh*	SAOUHSC_00300	Lipase 2	2.3	1.5
*sspA*	SAOUHSC_00988	V8 protease	2.4	1.1
*aur*	SAOUHSC_02971	Aureolysin	1.7	1.2
*sspP*	SAOUHSC_02127	Staphopain A precursor	1.2	*0.2* ^b^
*sspB*	SAOUHSC_00987	Staphopain B	2.7	1.6
*psmβ1*	SAOUHSC_01135	Phenol soluble modulin beta 1	2.7	*−1.3* ^b^
*psmβ2*	SAOUHSC_01136	Phenol soluble modulin beta 2	3.1	*−1.4* ^b^
*spoVG*	SAOUHSC_00469	Regulatory protein SpoVG	2.1	*−0.2* ^b^
*sasC*	SAOUHSC_01873	Intercellular aggregation protein	*−0.1* ^b^	2.2

^*a*^ Values are shown as mean fold changes from three independent biological replicates. Statistical significance was assessed using linear regression via the *pcr* R package.

^*b*^ Values that are significant (*P* > 0.05 and fold change ≥1.5 or ≤−1.5). *sspP* (*P *= 0.77), *psmβ1* (*P *= 0.62), *psmβ2* (*P* = 0.53), *spoVG* (*P* = 0.5), *sasC *(*P *= 0.8). Italics indicate numbers that are not significantly different.

*S. aureus* produces an extensive repertoire of virulence factors (72). Previously, we demonstrated that a fully functional stringent response is required for virulence in *S. aureus* cutaneous infections (20). For this reason, we also compared the expression levels of genes encoding specific virulence factors between the WT and the *rsh*_syn_ mutant under stringent conditions (Table 2). After mupirocin treatment, there was 2.1-, 2.6-, and 2.5-fold upregulation of cytolytic toxins alpha toxin (*hla*) and *lukED,* respectively, in the WT, and *hla* and *lukD* were increased by 1.6- to 1.7-fold in the *rsh_syn_* mutant (Table 2). Alpha toxin is a major secreted cytolytic toxin that assembles into a heptameric pore, causing lysis of epithelial cells, lymphocytes (73), monocytes (73), and endothelial cells (74). It also plays a significant role in disease pathogenesis since *hla*-deficient mutants exhibit reduced virulence in animal models of cutaneous infection (75), pneumonia (76), peritonitis (77), and sepsis (74).

Mupirocin treatment also caused 5.5- and 2.3-fold upregulation of the lipase-1 and lipase-2 genes, respectively, which encode enzymes that degrade/evade host defenses. Upregulation was substantially suppressed in the *rsh_syn_* mutant. Interestingly, *S. aureus* is exposed to substantial amounts of bactericidal lipids in abscess tissues; thus, lipases are likely to play a crucial role in supporting bacterial persistence within the abscess environment (78, 79). Lipases are essential for peritoneal abscess and biofilm formation (80). In addition to these cytolytic toxins and lipases, mupirocin treatment led to an upregulation of the expression of major proteases, V8 protease (*sspA*), aureolysin (*aur*), and staphopain (*sspB*), and this is, in part, dependent on the SSR. V8 serine protease has been shown to cleave human immunoglobulin (81), while aureolysin degrades the antimicrobial peptide LL-3 (82). Staphopains are cysteine proteases that cleave elastin (83) and degrade fibrinogen and collagen (84). It seems possible that the induction of these genes under stringent conditions could be associated with increased invasiveness, suppression of host defense mechanisms, and dissemination to escape the local environment.

Interestingly, under stringent conditions, the genes encoding phenol-soluble modulins (PSMs), specifically toxins β1 and β2, which are critical virulence factors (85), were upregulated by 2.7- and 3.1-fold, respectively, in the WT, but not in the *rsh*_syn_ mutant. This suggests that these toxins are regulated by the SSR. Unlike other toxins, which are highly specific for certain cell types or host species, PSMs can lyse most eukaryotic cells (86). As a result, PSMs are major contributors to virulence in community-associated MRSA strains, and deletion of the PSM locus has been shown to attenuate abscess pathology in a murine skin infection model (87). Thus, our data suggest that the stringent response may regulate toxin production, possibly as a survival strategy during stress.

### DJK-5 altered the expression of *S. aureus* stringent response markers and suppressed methicillin-resistant *S. aureus-*induced toxicity

Substantial evidence indicates that DJK-5 reduces ppGpp levels (18, 19, 23, 88), a finding we confirmed in this study, and also promotes additional bacterial killing (23, 89) via a mechanism yet to be elucidated. Using ^31^P-NMR spectroscopy to assess intracellular ppGpp levels, 64 μg/mL of DJK-5 reduced ppGpp levels by 26%–27% (integrating the peaks at 0.5–1 ppm and 1–2 ppm, respectively) compared to untreated cells. Increasing the DJK-5 concentration to 96 μg/mL led to a slight increase in the reduction in ppGpp levels, with a 30% reduction in signal between 1 and 2 ppm (Fig. S2).

Such ppGpp-targeted peptides can inhibit biofilm formation, infection, and virulence in *S. aureus* and other species (18–20). To further elucidate the mechanisms underlying this stringent response-targeted therapy, we investigated the effect of DJK-5 on hallmark stringent response genes. DJK-5 was administered to mupirocin-induced (i.e., stringently stressed, amino acid-limited) cells, and gene expression levels were analyzed using qRT-PCR. DJK-5 significantly reduced the induction of several stringent response-induced genes, including BCAA transporters (*brnQ1*), BCAA synthesis genes (*ilvC*), biofilm-associated genes (*ica*, *fnbA*), and virulence/protease genes (*psm*, *aur*, *sspA*), as measured by qRT-PCR (Table 3; Table S6).

**TABLE 3:** Effect of DJK-5 on mupirocin-induced stringent response marker genes measured by qRT-PCR^*a*^^,^^*b*^

Gene	Treatment	log2FC (95% CI)
*aur*	Mupirocin	2.30 (1.28–3.32)
*aur*	Mupirocin + DJK-5	−2.74 (−4.06 to −1.24)
*brnQ1*	Mupirocin	5.67 (4.74–6.60)
*brnQ1*	Mupirocin + DJK-5	−3.17 (−4.32 to −1.81)
*fnbA*	Mupirocin	4.60 (2.98–6.23)
*fnbA*	Mupirocin + DJK-5	−2.57 (−3.05 to −0.34)
*ica*	Mupirocin	3.21 (2.74–3.69)
*ica*	Mupirocin + DJK-5	−4.02 (−4.69 to −3.32)
*ilvC*	Mupirocin	5.47 (4.46–6.48)
*ilvC*	Mupirocin + DJK-5	−2.62 (−4.05 to −1.19)
*psm*	Mupirocin	1.22 (0.66–1.79)
*psm*	Mupirocin + DJK-5	−2.00 (−2.80 to −1.20)
*sspA*	Mupirocin	3.01 (1.94–4.08)
*sspA*	Mupirocin + DJK-5	−2.58 (−4.10- to −1.06)

^*a*^ Values are shown as log2 fold change relative to untreated control from three independent biological replicates. Positive values indicate upregulation and negative values indicate downregulation.

^*b*^ Confidence intervals were calculated by log2 transformation of fold-change confidence intervals derived from ΔΔCt variance across biological replicates (calculated using linear regression via the *pcr* R package).

**Fig 3:**
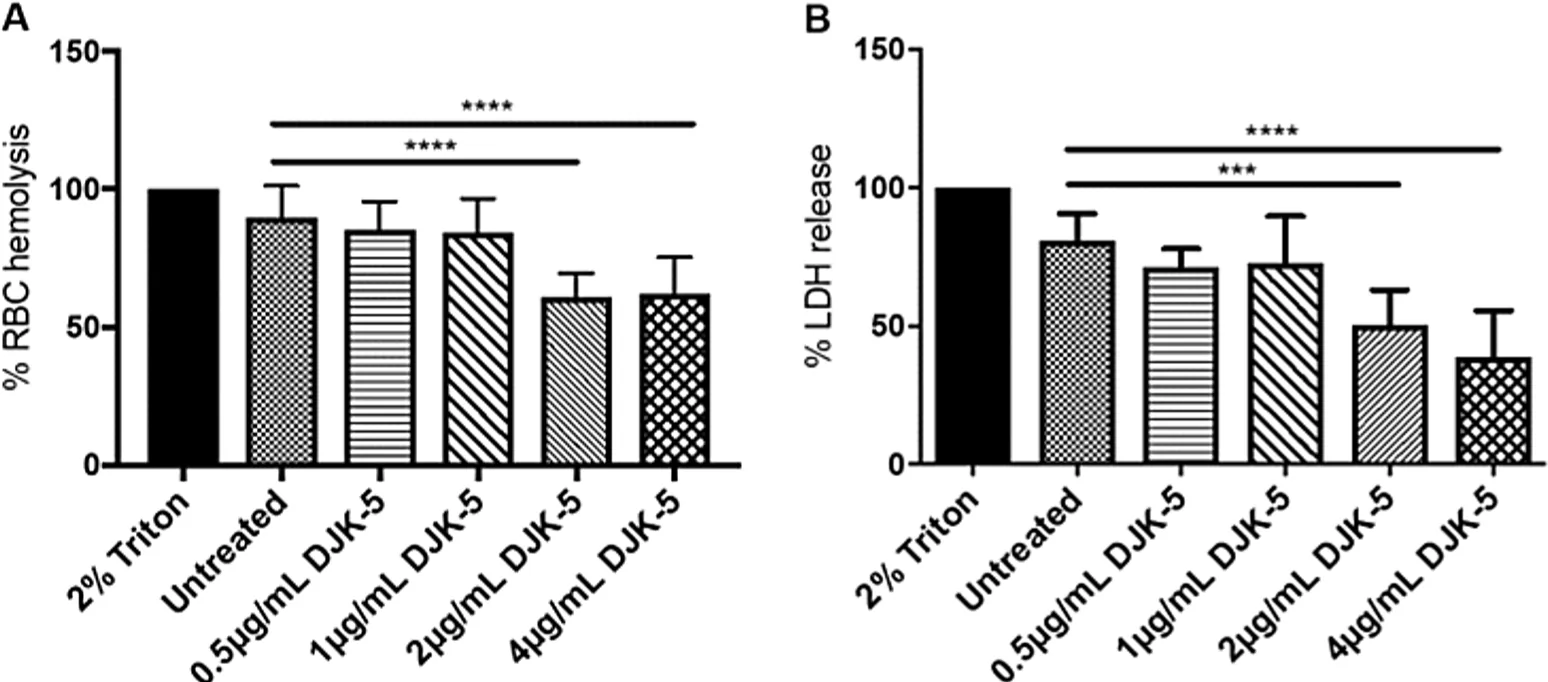
DJK-5 suppressed cytotoxicity of MRSA toward eukaryotic cells. (**A**) RBC lysis caused by MRSA filtrates harvested from bacteria treated with 0.5–4 μg/mL DJK-5 overnight. (**B**) LDH release of HBEC caused by MRSA filtrates harvested from bacteria treated with 0.5–4 μg/mL DJK-5 overnight. Data are shown as mean ± SD from three independent biological replicates; for the RBC assay, each replicate used blood from a different donor. Statistical significance was determined by one-way ANOVA with Dunnett’s multiple comparisons test relative to the untreated control. ***P < 0.001, ****P < 0.0001.

Stringent response mutants have previously been shown to be impaired in hemolytic and cytolytic activity against eukaryotic cells in gram-negative *P. aeruginosa* (23). Since peptide treatment suppressed the expression of several membrane-damaging toxins, including PSMs, the ability of the peptide to inhibit *S. aureus*-induced toxicity toward eukaryotic cells was further examined. An invasive CA-MRSA isolate, USA300, was grown overnight in the presence of sub-effective concentrations of DJK-5 (0.5 to 4 μg/mL), and the supernatants were incubated with red blood cells or human bronchial epithelial cells. Supernatants of USA300 treated with either 2 or 4 μg/mL of DJK-5 were ~35% less lytic toward red blood cells (Fig. 3A). They induced 50%–65% less LDH release from human bronchial epithelial cells (Fig. 3B), when compared to release into the supernatants from untreated bacteria. Therefore, these results indicate that interference with the stringent response can reduce cytotoxicity toward eukaryotic cells. While we cannot exclude the possibility that DJK-5 modulates the expression of additional toxins not examined in this study, the combined effect of the peptide on various virulence determinants likely contributes to its reduced toxicity toward eukaryotic cells, and likewise to the reduced severity of MRSA cutaneous abscess formation previously observed (20).

These findings are also consistent with our recent work showing that DJK-5 can enhance antibiotic activity in polymicrobial biofilm settings (22). In that study, DJK-5 increased *S. aureus* susceptibility to colistin in *P. aeruginosa–S. aureus* co-biofilms and showed combination activity in a murine subcutaneous biofilm-like abscess model (22), supporting a broader role for DJK-5 as an antibiofilm sensitizing agent under infection-relevant conditions. Together, these studies suggest that DJK-5 may have utility both as a biofilm-active sensitizer and as a tool to perturb stringent-response-associated virulence programs, although the relative contribution of these activities is likely to depend on biological context. The translational development of DJK-5 remains at an early stage. DJK-5 is an all-D antibiofilm peptide, a class generally associated with improved protease resistance, but important development questions remain unresolved, including pharmacokinetics, tissue distribution, route of administration, and access to infected tissues. Previous work has also explored formulation strategies, including hyaluronic acid-based nanogels (90), to improve *in vivo* compatibility. These considerations suggest that local or formulation-assisted delivery may be more realistic near-term applications than systemic use, and additional pharmacological studies will be required to define the clinical potential of DJK-5 more clearly.

### Conclusion

The stringent response plays a central role in *S. aureus* adaptation to stress, regulating both intracellular gene expression and the composition of EV-associated RNA cargo. Our findings reveal that disruption of (p)ppGpp synthesis profoundly alters the sRNA cargo of EVs. Among the annotated EV-associated sRNAs, SprX2 emerged as a candidate of interest, but its contribution to the observed phenotypes remains to be studied. Likewise, other altered EV-associated sRNAs may also contribute to these effects. Pharmacological inhibition with DJK-5 reduced the intracellular ppGpp signal, suppressed stringent-response-associated virulence genes, and reduced MRSA-mediated cytotoxicity. These results support a model in which stringent-response inhibition may attenuate stress adaptation and virulence while also altering EV-associated RNA cargo. This approach may enhance antimicrobial efficacy against recalcitrant MRSA infections.

## Data Availability

The raw FASTQ files for the sRNA-seq analysis are available under NCBI SRA (NCBI Sequence Read Archive) BioProject PRJNA1301528. The raw FASTQ files from the bulk RNA-seq analysis are available under NCBI SRA BioProject PRJNA433003.

## SUPPLEMENTAL MATERIAL

Table S1 spectrum.00359-26-s0001.xlsx Primer sequences.

Supplemental Figures spectrum.00359-26-s0002.docx Fig. S1 and S2.

Supplemental Text spectrum.00359-26-s0003.docx Inhibition of leucine incorporation into proteins using mupirocin altered the *S. aureus* transcriptome.

Supplemental Tables spectrum.00359-26-s0004.xlsx Tables S2 to S4.

Table S5 spectrum.00359-26-s0005.xlsx Processed EV-associated sRNA annotations and genotype-specific candidate sRNAs detected in extracellular vesicles from wild-type and Δ*rshsyn S. aureus*.

Table S6 spectrum.00359-26-s0006.xlsx ΔΔCt-based qPCR analysis of gene expression changes following Mupirocin alone and in combination with DJK5 treatment.

ASM does not own the copyrights to Supplemental Material that may be linked to, or accessed through, an article. The authors have granted ASM a non-exclusive, world-wide license to publish the Supplemental Material files. Please contact the corresponding author directly for reuse.
