# Supplementary material for: Targeting the stringent response alters gene expression and extracellular vesicle RNA in Staphylococcus aureus
Source: Microbiol Spectr. 2026 Jun 15;14(7):e00359-26. doi: 10.1128/spectrum.00359-26 (PMC13340242; doi:10.1128/spectrum.00359-26)
Supplement: Supplemental Figures — Fig. S1 and S2. [file spectrum.00359-26-s0002.docx]

**Supplementary Figures**

**
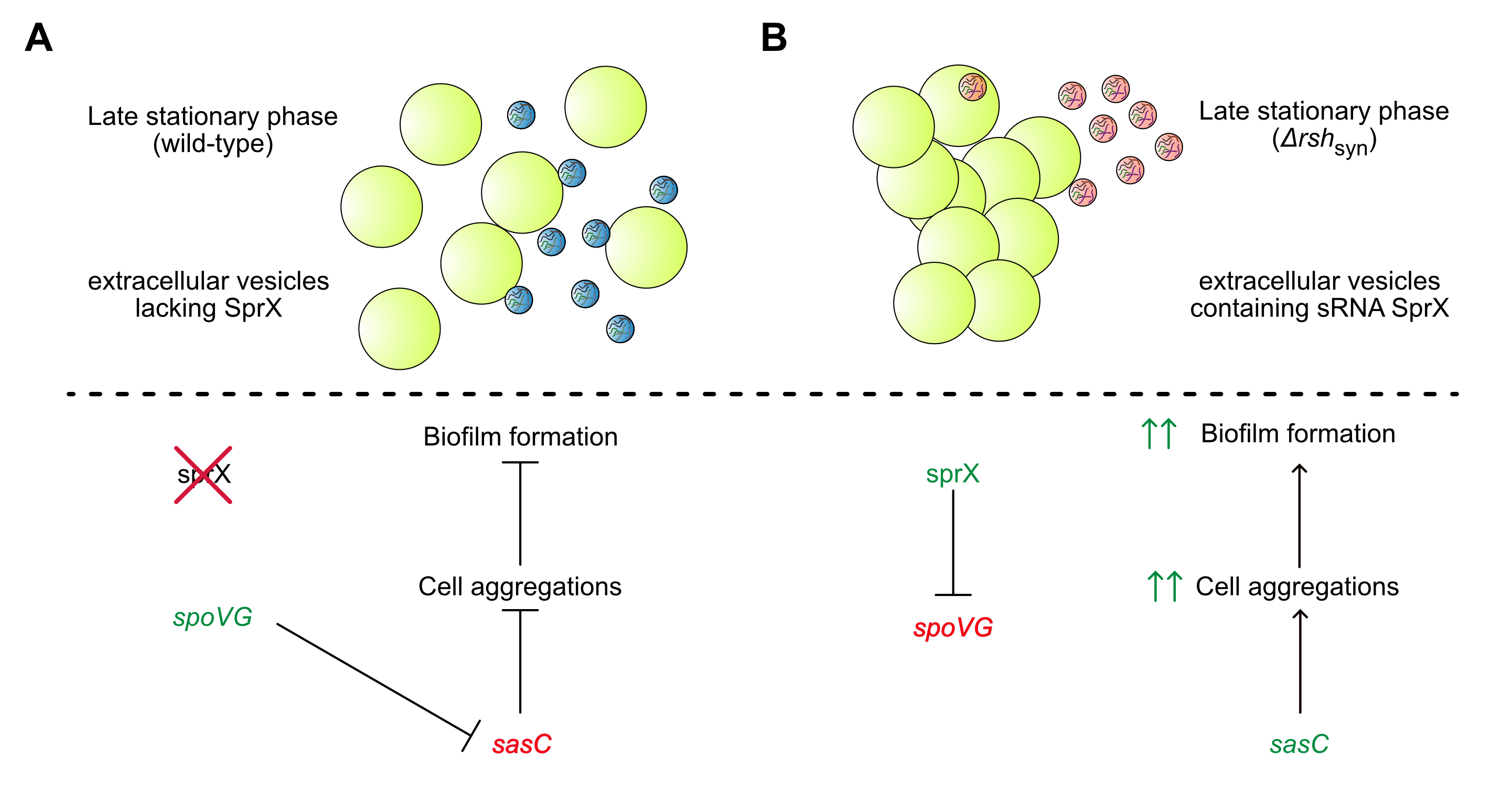
**

**Supplementary Figure S1: Working model for a potential SprX–SpoVG–SasC axis associated with altered aggregation/biofilm phenotypes in the *rsh*_syn_ mutant strain.** Wild-type conditions (A) vesicles lacking *sprX* (blue circles) allow for SpoVG to inhibit *sasC*, this is supported by *spoVG* being upregulated when stringent response is induced using mupirocin in the wild-type (**Table 2**). In the Δ*rsh*_syn_ strain (B), the small RNA *sprX* is present in extracellular vesicles (red circles) during late stationary phase, preventing spoVG protein accumulation and allowing *sasC* expression, leading to increased cell aggregation and biofilm formation. This is supported by *sasC* being upregulated when induced into stringent response with mupirocin (**Table 2**).

**
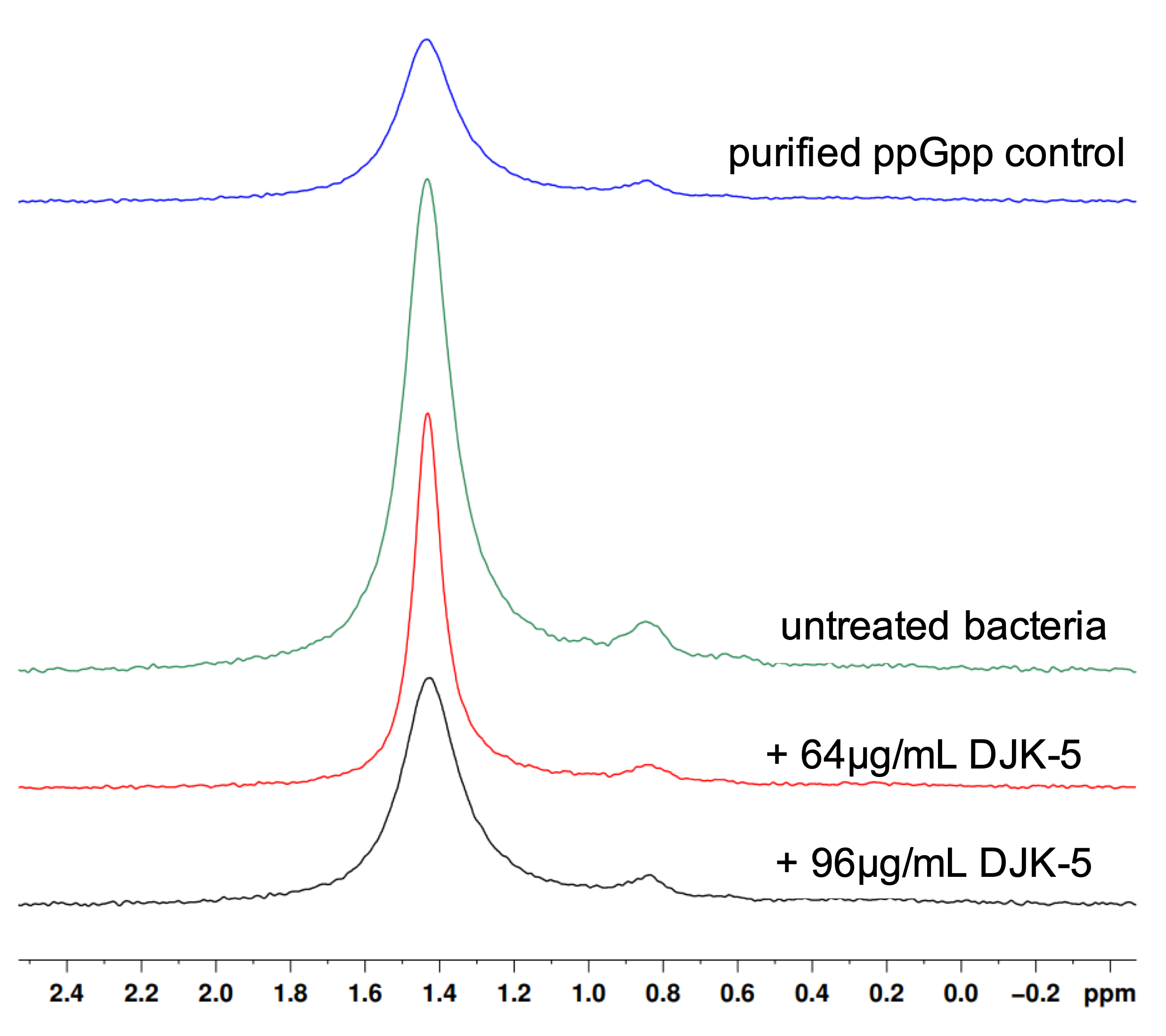
**

**Supplementary Figure S2: DJK-5 binding and degradation of ppGpp.** ^31^P nucleic magnetic resonance (NMR) spectra. Blue line: purified ppGpp reference, Green: untreated *S. aureus* cells, Red: *S. aureus* treated with 64 µg/mL DJK-5, Black: *S. aureus* treated with 96 µg/mL DJK-5.
