# Supplementary material for: Targeting the stringent response alters gene expression and extracellular vesicle RNA in Staphylococcus aureus
Source: Microbiol Spectr. 2026 Jun 15;14(7):e00359-26. doi: 10.1128/spectrum.00359-26 (PMC13340242; doi:10.1128/spectrum.00359-26)
Supplement: Supplemental Text — Inhibition of leucine incorporation into proteins using mupirocin altered the S. aureus transcriptome. [file spectrum.00359-26-s0003.docx]

**Supplementary Text. Inhibition of isoleucyl-tRNA synthetase by mupirocin altered the *S. aureus* transcriptome**

Mupirocin is an agent that inhibits isoleucyl tRNA synthetase and inhibits chain elongation in protein synthesis ^1^. Treatment with mupirocin caused significant (corrected *p*-value ≤ 0.05) 1.5-fold or greater expression changes in 1,722 genes (854 up-, 868 down-regulated) in the wild-type (WT) and 1,519 genes (796 up-, 723 down-regulated) in the mutant, representing 60% and 53% of the gene content in the *S. aureus* genome respectively when compared to their respective transcriptomes under untreated conditions. Comparison of WT and mutant changes revealed that the stringent response was responsible for most of the mupirocin-regulated changes, with 805 genes being relatively downregulated by 1.5 to 799-fold in the *rsh_syn_* mutant and 713 genes being upregulated by 1.5 to 137-fold in the mutant. The number of gene expression changes we observed was considerably greater than previous studies (showing 248 genes ^2^, ~600 genes ^3^ or 869 genes ^4^ dysregulated by mupirocin treatment), likely reflecting the use of different methods (e.g. microarrays for the first two studies) and/or parameters.

Differentially expressed (DE) genes were mapped to biological pathways (KEGG) to identify significantly enriched upregulated and downregulated pathways (**Appendix A-B**). Further pathway enrichment analysis was performed using SIGORA (**Appendix C**). Overall, this analysis demonstrated that mupirocin caused a significant upregulation in amino acid biosynthesis, secondary metabolite biosynthesis and metabolic pathways, as well as a suppression of genes involved in translation in WT. In the mutant, ribosomal and transport pathways were upregulated, while carbohydrate metabolism and secondary metabolite biosynthesis were suppressed (**Appendix C**). We examined some of these categories in greater detail, as well as genes involved in transport, adhesion, and virulence.

In the WT, mupirocin caused an upregulation of more than 50% of the genes found in amino acid biosynthesis pathways. Most notably, genes involved in the biosynthesis of branched-chain amino acids (BCAA) isoleucine, leucine, and valine (ILV), which *S. aureus* requires for the synthesis of proteins and membrane branched-chain fatty-acids ^5^. The system is part of a nine-gene operon *ilvDBHC*-*leuABCD*-*ilvA* (SAOUHSC_02281-02289), and genes were on average 1,045-fold upregulated after mupirocin treatment, representing the most upregulated genes in the dataset (**Appendix D; Supplementary Table S2**). The upregulation of the ILV operon was dependent on ppGpp as it was not differentially expressed in the *rsh*_syn_ mutant after mupirocin treatment (**Supplementary Table S3**). Interestingly, BCAAs also activate the major transcriptional regulator CodY, which regulates genes involved in environmental adaptation, small RNA signalling molecules, metabolism, and virulence ^6-11^.

Together with increased induction of BCAA biosynthesis pathways, mupirocin also caused an upregulation of genes involved in basic amino acid (lysine and histidine) biosynthesis pathways. L-lysine is produced from aspartate through the diaminopimelate (DAP) pathway ^12^. Various genes encoding enzymes that catalyze this pathway (*lysC*, *asd*, *dapA*, *dapB*, *dapD* and *dapL*) were 20 to 330-fold upregulated. Likewise, histidine biosynthesis genes (*hisA*, *hisB*, *hisD*, *hidF*, *hisG*, *hisH*, *hisZ*) were upregulated in the WT by 4- to 200-fold after mupirocin treatment when compared to untreated conditions. Three methionine biosynthesis operons (*metEFCI*, *metQ2P2N2*, and *metN1P1Q1*) and the threonine *thrD-hom-thrCB* biosynthesis operon were highly upregulated on average 24-fold, 110-fold, 1,300-fold, and 60-fold, respectively. As with BCAA, the biosynthesis of lysine, histidine, methionine, and threonine was not significantly induced in the *rsh*_syn_ mutant, indicating that these genes were regulated by ppGpp and the stringent response.

In response to amino acid deprivation, 82 genes involved in translation were downregulated by 1.5 to 13-fold in WT *S. aureus*, including most of the genes encoding the small and large subunit ribosomal proteins (i.e., *rpsA-T*, *rpmA-H* and *rplA-T*) as well as many genes encoding aminoacyl-tRNA synthetases (e.g., *serS*, *hisS* and *tyrS*) (**Appendix D; Supplementary Table S2**). Interestingly, in the *rsh*_syn_ mutant, genes encoding 30S and 50S ribosomal proteins were upregulated after mupirocin treatment, while genes encoding aminoacyl-tRNA synthetases and 19 of the 22 tRNA-encoding genes were downregulated. This likely reflects the importance of RSH in regulating the coordination of the translational apparatus to compensate for the loss of protein synthesis capacity under amino acid limitation caused by mupirocin.

Along with the heightened expression of BCAA synthesis genes, mupirocin treatment caused a strong upregulation in the BCAA transporter BrnQ1 by 17.1-fold in the WT. Like BCAA synthesis, this was an RSH-dependent event, and induction of this gene was not observed in the *rsh*_syn_ mutant. Amino acid deprivation also caused upregulation of the conserved oligopeptide permease transport system Opp-3 in the WT, which was consistent with previous data ^2^. As with other ATP-binding cassette (ABC) superfamilies, Opp importers are typically composed of five subunits, OppA, OppB, OppC, OppD and OppF, which function as a complex to import peptides from the external environment. As such, Opp-3 has been shown to be involved in nutrient uptake as well as many diverse processes such as quorum sensing, antimicrobial peptide resistance and bacterial colonization ^13^. The *opp-3A* gene, which expresses a protein involved in the capture of peptides from the external environment, was 520-fold upregulated, while the other genes within the *opp* operon were on average 330-fold upregulated. Interestingly, the *opp3* system was not strongly upregulated in the *rsh* synthase mutant, indicating that ppGpp regulated the expression of this system, possibly through indirect influence of CodY; a transcription factor previously shown to bind to the opp promoter site^6^. Overall, it appears that the cells requirement for BCAAs when depleted by mupirocin treatment can be offset by upregulating amino acid acquisition from the environment^7^ by enhancing BCAA and peptide transport through *brnQ1* and *opp3*.

**References**

1 Crosse, A. M., Greenway, D. L. & England, R. R. Accumulation of ppGpp and ppGp in *Staphylococcus aureus* 8325-4 following nutrient starvation. *Lett. Appl. Microbiol.* **31**, 332-337 (2000).

2 Anderson, K. L., Roberts, C., Disz, T., Vonstein, V., Hwang, K., Overbeek, R., Olson, P. D., Projan, S. J. & Dunman, P. M. Characterization of the *Staphylococcus aureus* heat shock, cold shock, stringent, and SOS responses and their effects on log-phase mRNA turnover. *J. Bacteriol.* **188**, 6739-6756 (2006). <https://doi.org/10.1128/JB.00609-06>

3 Reiss, S., Pané-Farré, J., Fuchs, S., François, P., Liebeke, M., Schrenzel, J., Lindequist, U., Lalk, M., Wolz, C., Hecker, M. & Engelmann, S. Global analysis of the *Staphylococcus aureus* response to mupirocin. *Antimicrobial Agents and Chemotherapy* **56**, 787-804 (2012). <https://doi.org/10.1128/AAC.05363-11>

4 AlHoufie, S. T. S. & Foster, H. A. Effects of sub-lethal concentrations of mupirocin on global transcription in *Staphylococcus aureus* 8325-4 and a model for the escape from inhibition. *J. Med. Microbiol.* **65**, 858-866 (2016). <https://doi.org/10.1099/jmm.0.000270>

5 Lei, T., Yang, J., Zheng, L., Markowski, T., Witthuhn, B. A. & Ji, Y. The essentiality of *Staphylococcal* Gcp is independent of its repression of branched-chain amino acids biosynthesis. *PLOS ONE* **7**, e46836 (2012). <https://doi.org/10.1371/journal.pone.0046836>

6 Majerczyk, C. D., Dunman, P. M., Luong, T. T., Lee, C. Y., Sadykov, M. R., Somerville, G. A., Bodi, K. & Sonenshein, A. L. Direct targets of CodY in *Staphylococcus aureus*. *J. Bacteriol.* **192**, 2861-2877 (2010). <https://doi.org/10.1128/JB.00220-10>

7 Kaiser, J. C., King, A. N., Grigg, J. C., Sheldon, J. R., Edgell, D. R., Murphy, M. E. P., Brinsmade, S. R. & Heinrichs, D. E. Repression of branched-chain amino acid synthesis in *Staphylococcus aureus* is mediated by isoleucine via CodY, and by a leucine-rich attenuator peptide. *PLoS Genet.* **14**, e1007159 (2018). <https://doi.org/10.1371/journal.pgen.1007159>

8 Menard, G., Silard, C., Suriray, M., Rouillon, A. & Augagneur, Y. Thirty Years of sRNA-Mediated Regulation in *Staphylococcus aureus*: From Initial Discoveries to In Vivo Biological Implications. *Int J Mol Sci* **23** (2022). <https://doi.org/10.3390/ijms23137346>

9 Augagneur, Y., King, A. N., Germain-Amiot, N., Sassi, M., Fitzgerald, J. W., Sahukhal, G. S., Elasri, M. O., Felden, B. & Brinsmade, S. R. Analysis of the CodY RNome reveals RsaD as a stress-responsive riboregulator of overflow metabolism in *Staphylococcus aureus*. *Mol Microbiol* **113**, 309-325 (2020). <https://doi.org/10.1111/mmi.14418>

10 Majerczyk, C. D., Dunman, P. M., Luong, T. T., Lee, C. Y., Sadykov, M. R., Somerville, G. A., Bodi, K. & Sonenshein, A. L. Direct targets of CodY in *Staphylococcus aureus*. *J Bacteriol* **192**, 2861-2877 (2010). <https://doi.org/10.1128/JB.00220-10>

11 Stenz, L., Francois, P., Whiteson, K., Wolz, C., Linder, P. & Schrenzel, J. The CodY pleiotropic repressor controls virulence in Gram-positive pathogens. *FEMS Immunol Med Microbiol* **62**, 123-139 (2011). <https://doi.org/10.1111/j.1574-695X.2011.00812.x>

12 Rodionov, D. A., Vitreschak, A. G., Mironov, A. A. & Gelfand, M. S. Regulation of lysine biosynthesis and transport genes in bacteria: yet another RNA riboswitch? *Nucleic Acids Res* **31**, 6748-6757 (2003). <https://doi.org/10.1093/nar/gkg900>

13 Diep, B. A. & Otto, M. The role of virulence determinants in community-associated MRSA pathogenesis. *Trends in microbiology* **16**, 361-369 (2008). <https://doi.org/10.1016/j.tim.2008.05.002>

**Appendix**


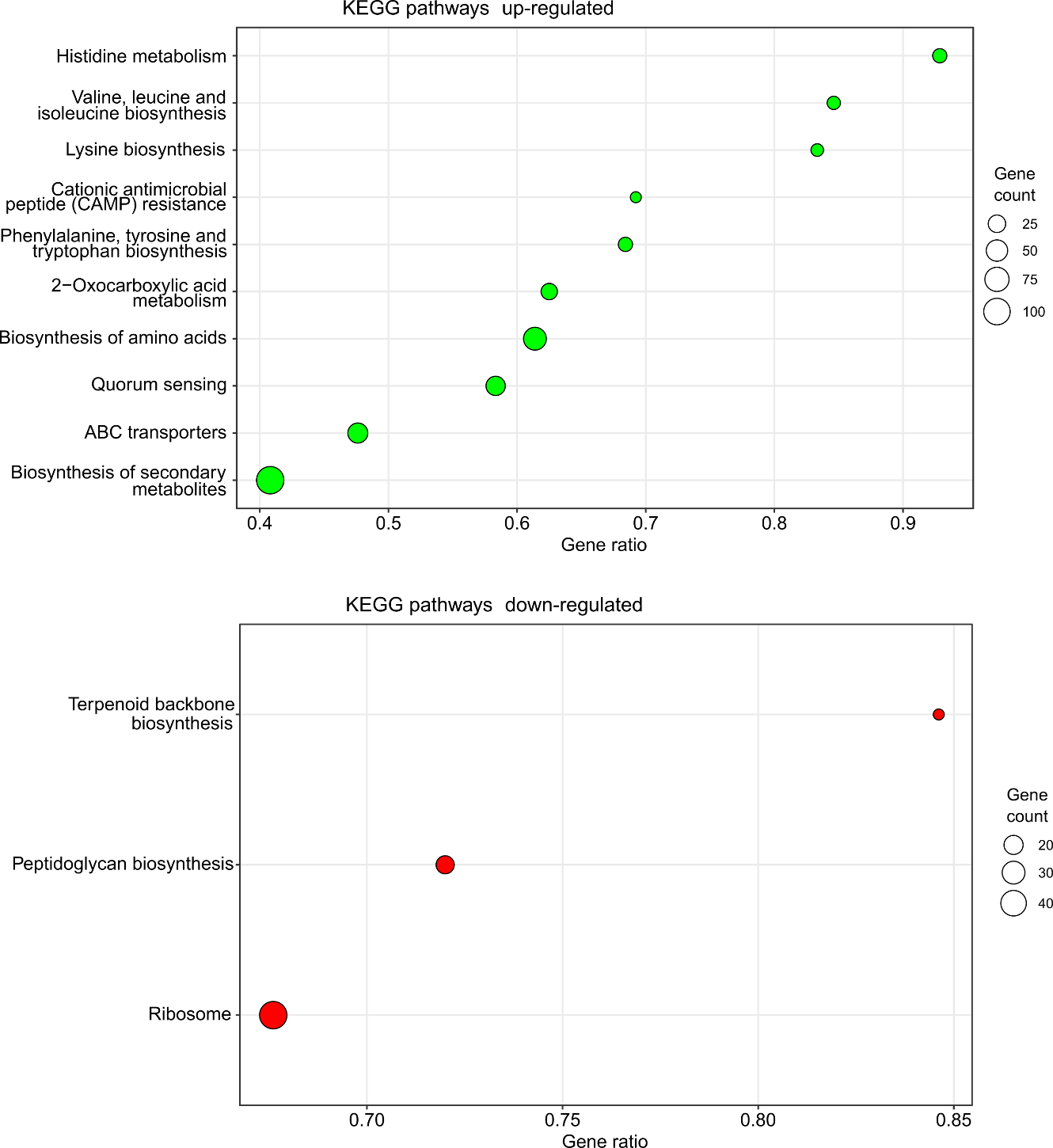


**Appendix A: Gene ratio visualization of significantly enriched KEGG pathways for WT *S. aureus* treated with mupirocin.** Significantly upregulated pathways (top, green), and significantly downregulated pathways (bottom, red). Gene ratio represents proportion of genes belonging to their respective pathway which are differentially expressed.


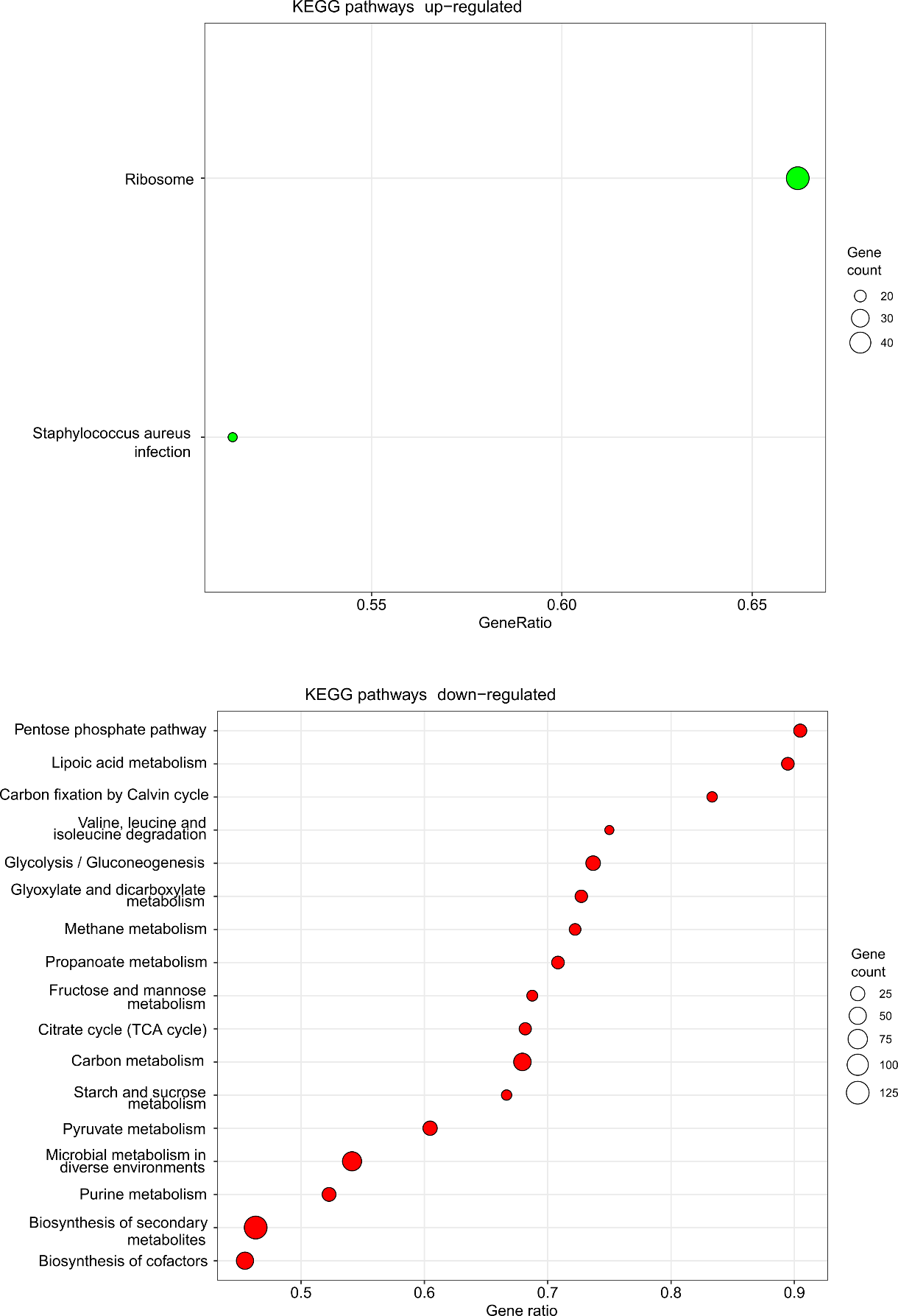


**Appendix B: Gene ratio visualization of significantly enriched KEGG pathways for *rsh_syn_* mutant *S. aureus* treated with mupirocin.** Significantly upregulated pathways (top, green), and significantly downregulated pathways (bottom, red). Gene ratio represents proportion of genes belonging to their respective pathway which are differentially expressed.

**Appendix C: Enriched pathways dysregulated by mupirocin treatment in *S. aureus* WT and *rsh_syn_* mutant.**

| **KEGG Pathway ID** | **Description** | **DE genes** | **Genes in pathway** | **Adjusted**  ***p-*value** |
| --- | --- | --- | --- | --- |
| **Upregulated Pathways in WT** | | | | |
| sao01230 | Biosynthesis of amino acids | 60 | 105 | 2.2 x 10^-10^ |
| sao01110 | Biosynthesis of secondary metabolites | 97 | 223 | 1.4 x 10^-07^ |
| sao01130 | Biosynthesis of antibiotics | 75 | 167 | 1.6 x 10^-06^ |
| sao00340 | Histidine metabolism | 13 | 15 | 5.5 x 10^-06^ |
| sao00290 | Valine, leucine and isoleucine biosynthesis | 11 | 13 | 5.1 x 10^-05^ |
| sao01100 | Metabolic pathways | 161 | 453 | 3.1 x 10^-05^ |
| sao01210 | 2-Oxocarboxylic acid metabolism | 15 | 20 | 2.6 x 10^-05^ |
| sao00300 | Lysine biosynthesis | 10 | 13 | 4.9 x 10^-04^ |
| **Downregulated Pathways in WT** | | | | |
| sao03010 | Ribosome | 49 | 71 | 1.8 x 10^-08^ |
| **Upregulated Pathways in *rsh_syn_* mutant** | | | | |
| sao03010 | Ribosome | 48 | 71 | 5.1 x 10^-14^ |
| sao02010 | ABC transporter | 42 | 102 | 5.0 x 10^-04^ |
| **Downregulated Pathways in *rsh_syn_* mutant** | | | | |
| sao01100 | Metabolic pathways | 188 | 453 | 1.6 x 10^-09^ |
| sao01200 | Carbon metabolism | 49 | 82 | 6.3 x 10^-08^ |
| sao00010 | Glycolysis/Gluconeogenesis | 28 | 40 | 5.8 x 10^-07^ |
| sao01130 | Biosynthesis of antibiotics | 88 | 167 | 1.3 x 10^-07^ |
| sao01110 | Biosynthesis of secondary metabolites | 99 | 223 | 6.9 x 10^-06^ |
| sao01120 | Metabolism in diverse environments | 66 | 130 | 1.5 x 10^-06^ |
| sao00030 | Pentose phosphate pathway | 17 | 20 | 1.2 x 10^-06^ |
| sao00620 | Pyruvate metabolism | 24 | 40 | 1.9 x 10^-04^ |
| sao00020 | Citrate cycle (TCA) | 15 | 22 | 4.9 x 10^-04^ |


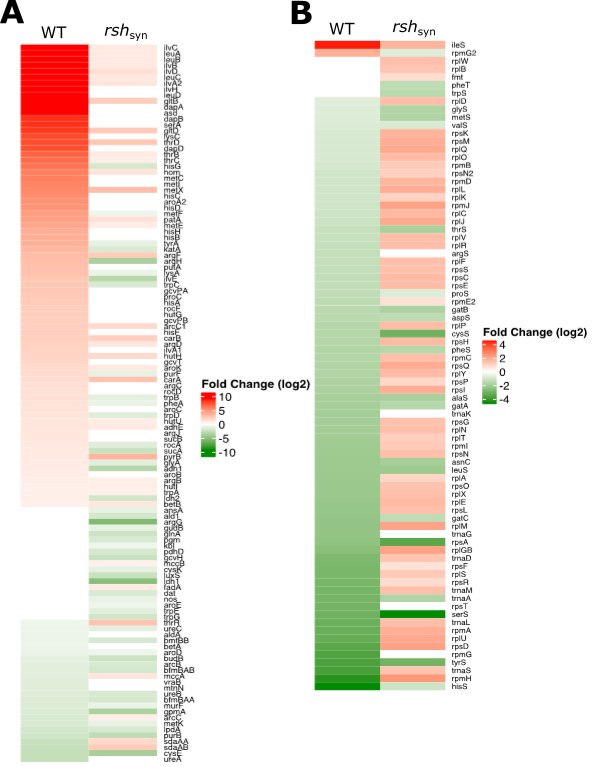


**Appendix D: Differentially expressed genes induced by mupirocin treatment show strong groupings into metabolic functions.** Genes dysregulated by mupirocin treatment in the WT (left column) and *rsh_syn_* mutant (right column). (**A**) Genes associated with amino acid metabolism. (**B**) Genes related to translation.
